# Hæmoglobinuria

**Published:** 1880-10

**Authors:** 


HÆMOGLOBINURIA.—District Veterinary Surgeon Loesch, of Neustadt, Germany, observed this disease in seven horses last December. One of the animals was seriously affected, the others suffered in a less degree. All finally recovered. The symptoms were the same in all essential particulars: unexpected and sudden seizure during work, after the animals had been stabled rather a long time, severe colics, profuse sweating, followed by paralysis and anæthasia of extremities, a considerably accelerated respiration, a pulse rate of 60 to 70, rectal temperature of 39·6° C, 40·2°, difficulty in passing water and the voiding of large quantities of a peculiar, oily, dark brown, turbid urine, of penetrating odor; his urine also contained large quantities of albumen. The disease lasted from 7 to 14 days. In two cases the rectal mucus membrane showed croupous deposits. The convalescing animals also evinced painful sensations during the passage of the soft excrements. Tenesmus also appeared to be present. Slimy, oily clysters afforded some relief. The treatment consisted of withdrawal of urine by means of the cathater about every six hours, warm fomentations, tepid injections, and several doses of salicylate of soda (each dose about 1½ oz.—*Thierarztliche Mittheilungen*, April, 1880.

